# The Anticancer Drug Ellipticine Activated with Cytochrome P450 Mediates DNA Damage Determining Its Pharmacological Efficiencies: Studies with Rats, Hepatic Cytochrome P450 Reductase Null (HRN™) Mice and Pure Enzymes

**DOI:** 10.3390/ijms16010284

**Published:** 2014-12-25

**Authors:** Marie Stiborová, Věra Černá, Michaela Moserová, Iveta Mrízová, Volker M. Arlt, Eva Frei

**Affiliations:** 1 Department of Biochemistry, Faculty of Science, Charles University, Hlavova 2030, CZ-12843 Prague 2, Czech Republic; E-Mails: vera.cerna@natur.cuni.cz (V.Č.); michaela.moserova@natur.cuni.cz (M.M.); iveta.vranova@natur.cuni.cz (I.M.); 2 Analytical and Environmental Sciences Division, MRC-PHE Centre for Environmental & Health, King’s College London, 150 Stamford Street, London SE1 9NH, UK; E-Mail: volker.arlt@kcl.ac.uk; 3 Division of Preventive Oncology, National Center for Tumor Diseases, German Cancer Research Center (DKFZ), Im Neuenheimer Feld 280, 69120 Heidelberg, Germany, E-Mail: eva.frei@t-online.de

**Keywords:** anticancer drug ellipticine, cytochrome P450 mediated DNA-damage, covalent DNA adducts, enzymes metabolizing ellipticine *in vitro* and *in vivo*

## Abstract

Ellipticine is a DNA-damaging agent acting as a prodrug whose pharmacological efficiencies and genotoxic side effects are dictated by activation with cytochrome P450 (CYP). Over the last decade we have gained extensive experience in using pure enzymes and various animal models that helped to identify CYPs metabolizing ellipticine. In this review we focus on comparison between the *in vitro* and *in vivo* studies and show a necessity of both approaches to obtain valid information on CYP enzymes contributing to ellipticine metabolism. Discrepancies were found between the CYP enzymes activating ellipticine to 13-hydroxy- and 12-hydroxyellipticine generating covalent DNA adducts and those detoxifying this drug to 9-hydroxy- and 7-hydroellipticine *in vitro* and *in vivo*. *In vivo*, formation of ellipticine-DNA adducts is dependent not only on expression levels of CYP3A, catalyzing ellipticine activation *in vitro*, but also on those of CYP1A that oxidize ellipticine *in vitro* mainly to the detoxification products. The finding showing that cytochrome *b*_5_ alters the ratio of ellipticine metabolites generated by CYP1A1/2 and 3A4 explained this paradox. Whereas the detoxification of ellipticine by CYP1A and 3A is either decreased or not changed by cytochrome *b*_5_, activation leading to ellipticine-DNA adducts increased considerably. We show that (I) the pharmacological effects of ellipticine mediated by covalent ellipticine-derived DNA adducts are dictated by expression levels of CYP1A, 3A and cytochrome *b*_5_, and its own potency to induce these enzymes in tumor tissues, (II) animal models, where levels of CYPs are either knocked out or induced are appropriate to identify CYPs metabolizing ellipticine *in vivo*, and (III) extrapolation from *in vitro* data to the situation *in vivo* is not always possible, confirming the need for these animal models.

## 1. Introduction

**Figure 1:**
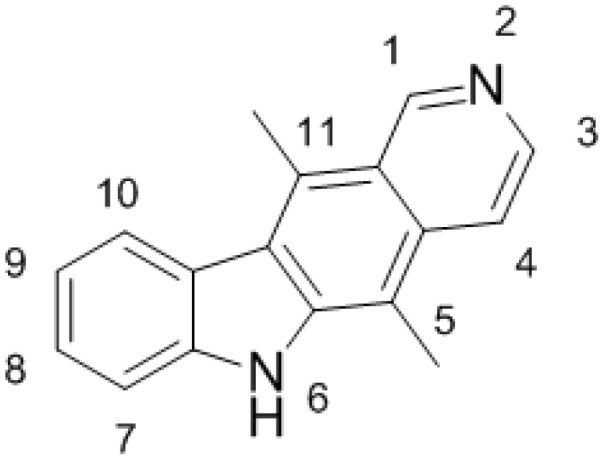
Ellipticine. Numbers 1–11 indicate locations of carbon and nitrogen atoms in the ellipticine molecule.

A plant alkaloid ellipticine (5,11-dimethyl-6H-pyrido[4,3-b]carbazole, Figure 1) found in several Apocynaceae plants and its derivatives are efficient anticancer compounds that function through multiple mechanisms participating in cell cycle arrest and the initiation of apoptosis (for a summary see: [1,2,3,4,5,6]). Ellipticine was found (I) to arrest cell cycle progression due to modulation of levels of cyclinB1 and Cdc2, and phosphorylation of Cdc2 in human mammary adenocarcinoma MCF-7 cells [7]; (II) to initiate apoptosis by several mechanisms such as formation of reactive oxygen species (ROS) inducing DNA damage, the activation of mitogen-activated protein kinases (MAPKs), release of cytochrome *c* and apoptosis-inducing factor (AIF) from the mitochondrial membrane, caspase activation as well as a caspase-independent pathway [8,9], triggering of Fas/Fas ligand pathway and modulation of proteins of the Bcl-2 family in several tumor cell lines [10]; (III) to disrupt mitochondrial function and (IV) to cause the apoptotic signaling that is amplified by cross-talk between the Fas death receptor and the mitochondrial apoptotic pathway (for a summary see: [3,4,7,8,9,10]).

Several studies also demonstrated that the p53 tumor suppressor protein is involved in ellipticine-mediated induction of cell cycle arrest and apoptosis [9,10,11,12,13,14,15,16,17,18,19,20]. Ellipticine inhibits p53 protein phosphorylation by a selective inhibition of CDK2 kinase in Lewis lung carcinoma and the human colon cancer cell line SW480 [11], and this effect on p53 correlated with cytotoxic activity of ellipticine [11]. Treatment of Saos 2 cells transfected with mutant p53 with ellipticine restored the transactivation function of p53, resulting in the induction of p53-responsive *p21^Waf^*^1^ and *MDM2* genes at protein levels and activation of a p53-responsive luciferase reporter [15]. The results found in the study of Sugikawa and coworkers [15] indicate that ellipticine induces a shift of mutant p53 conformation towards wild-type and this activity is not caused by its function as an inhibitor of topoisomerase II, which is one of the DNA-damaging effects of ellipticine (for a summary see [1,2,3,4,5,6]). More importantly, ellipticine can even activate mutant p53 and induces *p21^Waf^*^1^ and *MDM2* gene expression *in vivo*, in nude mouse tumor xenografts [16]. Moreover, Wang *et al.* [19] demonstrate that in mutant-p53 lymphoma cells, ellipticine-mediated reactivation of mutant p53 sensitizes these cells to treatment with further DNA-damaging drugs (*i.e.*, doxorubicin).

Ellipticine elevated the nuclear localization of endogenous p53 and exogenous mutant p53 in HCT116 colon cancer cells leading to transactivation of the p21 promoter. Nuclear localization of p53 is frequently the consequence of a genotoxic stress by compounds inducing DNA damage (*i.e.*, inhibitors of topoisomerase II) [21]. The ellipticine-mediated abundance of nuclear p53 was not associated with an increase in DNA double strand breaks. Therefore, this effect of ellipticine seems not to be dependent on the mechanism mediated by topoisomerase II inhibition, but on another genotoxic stress [22]. Further, ellipticine induced nuclear translocalization of p53 and of the serine/threonine kinase Akt (an enzyme providing a survival signal protecting cells from stress induced apoptosis) and recruitment of autophagosomes in human non-small cell lung cancer (NSCLC) epithelial cells A549 [18]. Akt-related cell death also occurred in p53-deficient cells with stable expression of exogenous p53. Hence, as a DNA-damaging agent, ellipticine is a regulator in autophagy-related cell death by cooperation of p53 and Akt [18]. Ellipticine also activates the p53 pathway in glioblastoma cells; its impact on these cancer cells depends on the p53 status [14]. In a U87MG glioblastoma cell line expressing wild-type p53, ellipticine provoked an early G0/G1 cell cycle arrest, whereas in a U373 cell line expressing a p53 mutant it induced arrest in S and G2/M phases of the cell cycle [14].

All studies investigating the mechanism of ellipticine antitumor action indicate complex pathways leading to cancer cell death by this drug. Chemotherapy-induced cell cycle arrest and induction of apoptosis were shown to frequently result from DNA damage caused by exposure to a variety of chemotherapeutics including ellipticine. In addition, genotoxic stress as a result of multiple DNA-damage increases levels of nuclear p53 [21,22], the tumor suppressor protein shown to be involved in ellipticine-mediated induction of cell cycle arrest and apoptosis [10,11,12,13,14,15,16]. These findings suggest that DNA damage by ellipticine is crucial for its cytotoxic effects.

## 2. DNA-Damaging Mechanisms of Ellipticine Cytotoxicity to Cancer Cells

The most important DNA-damaging mechanisms of ellipticine were considered to be intercalation into DNA [5,23,24,25,26] and inhibition of DNA topoisomerase II activity [5,27,28]. Recently, Andrews and co-workers [29] demonstrated, however, that ellipticine and some of its derivatives are potent and specific inhibitors of RNA polymerase I (Pol-I) transcription and that this Pol-I inhibition occurs by a p53- and topoisomerase II-independent mechanism. They found that the drug influences the assembly and stability of preinitiation complexes by targeting the interaction between promoter recognition essential transcription factor SL1 and the rRNA promoter. In addition, Ghosh *et al.* [30] showed that along with DNA intercalation and/or topoisomerase II inhibition, interaction with the telomeric DNA region and the resultant inhibition of telomerase activity might be an additional mode of action of ellipticine. Moreover, we showed that this antitumor agent also causes damage to the structural integrity of DNA through covalent binding, by forming covalent DNA adducts after its enzymatic activation with cytochrome P450 (CYP) or peroxidases [1,2,3,4,31,32,33,34,35,36,37,38,39,40]. Cytotoxicity of ellipticine in cells of several cancer lines sensitive to this drug such as HL-60 promyelocytic leukemia, T-cell leukemia CCRF-CEM, glioblastoma U87MG, neuroblastoma UKF-NB-3 and UKF-NB-4, thyroid cancer BHT-101, B-CPAP and 8505-C and breast adenocarcinoma MCF-7 corresponded to levels of ellipticine-derived DNA adducts generated after its enzymatic activation in most of these cell lines [41]. This indicates that covalent binding to DNA of reactive species generated by enzymatic bioactivation of ellipticine is one of the most important mechanisms responsible for ellipticine cytotoxicity in these cancer cells. The formation of ellipticine-DNA adducts ultimately forces cancer cells to initiate cell death signaling [9]. Based on these results, we suggest that ellipticine acts as a prodrug, which is metabolically activated to reactive species forming covalent DNA adducts causing genotoxic stress. Therefore, information on which enzymes are involved in the metabolism of ellipticine is critical to identify the pharmacological effects of ellipticine. Several *in vitro* and *in vivo* approaches have been developed to study the role of specific CYP and peroxidase enzymes in ellipticine metabolism.

**Figure 2:**
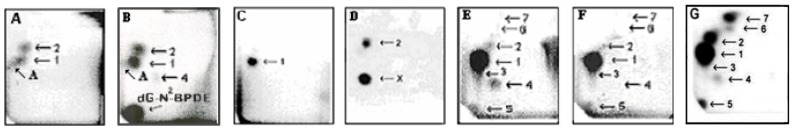
Autoradiographs of thin layer chromatography (TLC) maps of ^32^P-labeled digests of calf thymus DNA reacted with ellipticine activated by hepatic microsomes from wild-type (WT) mice (**A**), with those from Hepatic Cytochrome P450 Reductase Null (HRN) mice pre-treated with benzo[a]pyrene (BaP) (**B**), from calf thymus DNA reacted with 13-hydroxyellipticine (**C**) [31] or 12-hydroxyellipticine (**D**) [32] of DNA from livers of WT (**E**) and HRN (**F**) mice treated intraperitoneally (*i.p.*) with 10 mg ellipticine/kg body weight [48] and of DNA from liver of Wistar rats treated *i.p.* with 40 mg ellipticine per kilogram body weight (**G**) [33,37]. Analyses were performed by the nuclease P1 version of the ^32^P-postlabeling assay. Adduct spots 1–7 and A correspond to the ellipticine-derived DNA adducts. Besides adduct 2, another strong adduct (spot X in panel **D**), which was not found in any other activation systems or *in vivo* was generated by 12-hydroxyellipticine.

Over the past 10 years we have gained extensive experience in using the pure enzymes and the various animal models to study the ellipticine metabolism. During these studies, ellipticine was found to be oxidized by CYP and peroxidase enzymes to both electrophilic species forming covalent DNA adducts detected by ^32^P-postlabeling (Figure 2) and to detoxification metabolites [1,2,3,4,32,33,34,35,36,37,38,39,40,42,43,44,45,46,47,48,49,50,51]. Moreover, we characterized the reactions leading to their formation.

In this review we focus on comparison between the data found in the *in vitro* and *in vivo* studies investigating ellipticine metabolism and show a necessity of both approaches to obtain valid information on CYP enzymes participating in this process.

## 3. Metabolism of Ellipticine by Cytochromes P450 (CYPs), Peroxidases and Conjugation Enzymes *in Vitro*

**Figure 3:**
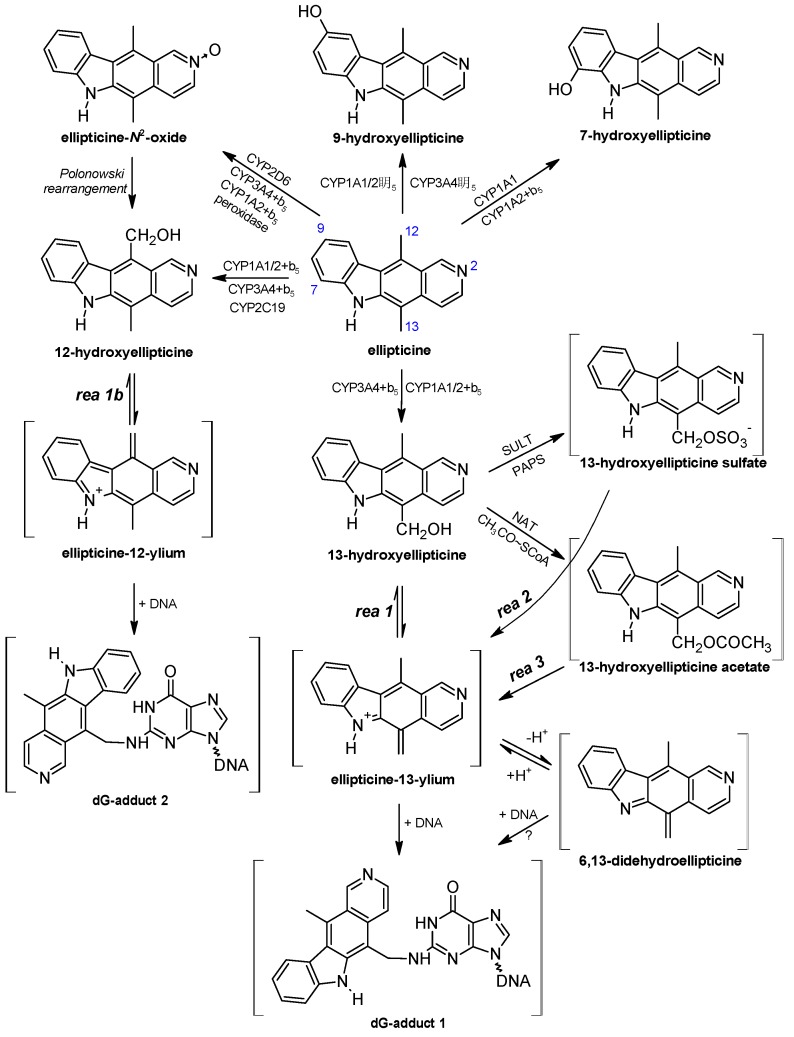
Ellipticine metabolism by CYPs showing the identified metabolites and those proposed to form DNA adducts. The compounds shown in brackets were not detected under the experimental conditions and/or are not yet structurally characterized. The CYP enzymes predominantly oxidizing ellipticine shown were identified in our previous studies [31,34,35,38,39]. Reactions 1, 2 and 3 lead to ellipticine-13-ylium from 13-hydroxyellipticine, 13-hydroxyellipticine sulfate and 13-hydroxyellipticine acetate, respectively, and rea 1b to ellipticine 12-ylium. ? indicates that the mechanisms of this reaction are not known.

Utilizing numerous *in vitro* systems such as subcellular microsomal fractions and cells in culture expressing CYPs, isolated CYPs reconstituted with other components of the mixed-function-oxidase system [NADPH:CYP reductase (POR), cytochrome *b*_5_], and recombinant CYPs, human, rat, rabbit, and mouse CYP enzymes were found to oxidize ellipticine. Ellipticine is oxidized to five metabolites, 7-hydroxy-, 9-hydroxy-, 12-hydroxy-, 13-hydroxyellipticine, and ellipticine *N*^2^-oxide (Figure 3), and at least two major ellipticine DNA-adducts were generated by these enzymatic systems [3,31,33,34,35,36,38,39,40,48,49]. 7-Hydroxy- and 9-hydroxyellipticine are efficiently excreted by experimental animals and considered to be the detoxification products of ellipticine [52,53]. But 9-hydroxyellipticine is also an efficient inhibitor of Pol-I transcription *in vitro* with IC_50_ values in cells in the nanomolar range [29], intercalates into DNA, and inhibits topoisomerase II activity [54,55,56]. It is therefore a pharmacologically important metabolite. 13-Hydroxy- and 12-hydroxyellipticine are the active metabolites, which spontaneously form ellipticine-13-ylium and ellipticine-12-ylium, which reacts with DNA to produce two major deoxyguanosine adducts (see adduct spots 1 and 2 in Figure 2 and the proposed structures of these DNA adducts in Figure 3) [2,3,4,31,32,34,35,36,39,40,49]. In addition, ellipticine *N*^2^-oxide is also considered a potent active ellipticine metabolite, since it converts to 12-hydroxyellipticine [31], by the Polonowski rearrangement [57] (Figure 3). All these results suggest that the enzymes activating or detoxifying ellipticine are crucial for its pharmacological effects. Thus, the identification of enzymes necessary for ellipticine metabolism is of great importance.

Using a variety of human recombinant CYPs, inhibitors of these enzymes in human hepatic microsomes and correlation analyses, the roles of individual CYPs in the formation of ellipticine metabolites was identified [31,34,35,40]. Human recombinant CYP1A1 and 1A2, followed by CYP1B1, are most effective in formation of 7-hydroxy- and 9-hydroxyellipticine detoxifying ellipticine (Figure 3) [31]. The active metabolite, 13-hydroxyellipticine, forming the ellipticine-DNA adduct 1 (Figure 2C), is generated predominantly by CYP3A4. Oxidation of ellipticine to another activation metabolite, 12-hydroxyellipticine, generating DNA adduct 2 (Figure 2D) is also catalyzed by CYP3A4, but more efficiently by CYP2C19. The *N*^2^-oxide of ellipticine is generated mainly by CYP2D6 beside CYP3A4 [31,34,35,39]. These results demonstrate that CYP3A4 is the most effective enzyme leading to ellipticine-DNA adducts 1 and 2, while adduct 2 is also generated by CYP2C19 and 2D6. Moreover, orthologous CYP enzymes of rats and mice catalyze formation of these metabolites and DNA adducts [1,2,3,4,36,38]. This indicates that these animals might be suitable models mimicking the fate of ellipticine in human.

Recently, we could show that levels of the DNA adduct formed by 13-hydroxyellipticine increased if this ellipticine metabolite was conjugated with sulfate or acetate by human sulfotransferases 1A1, 1A2, 1A3 and 2A1, or *N*,*O*-acetyltransferases 1 and 2 (Figure 3) [34,58].

**Figure 4:**
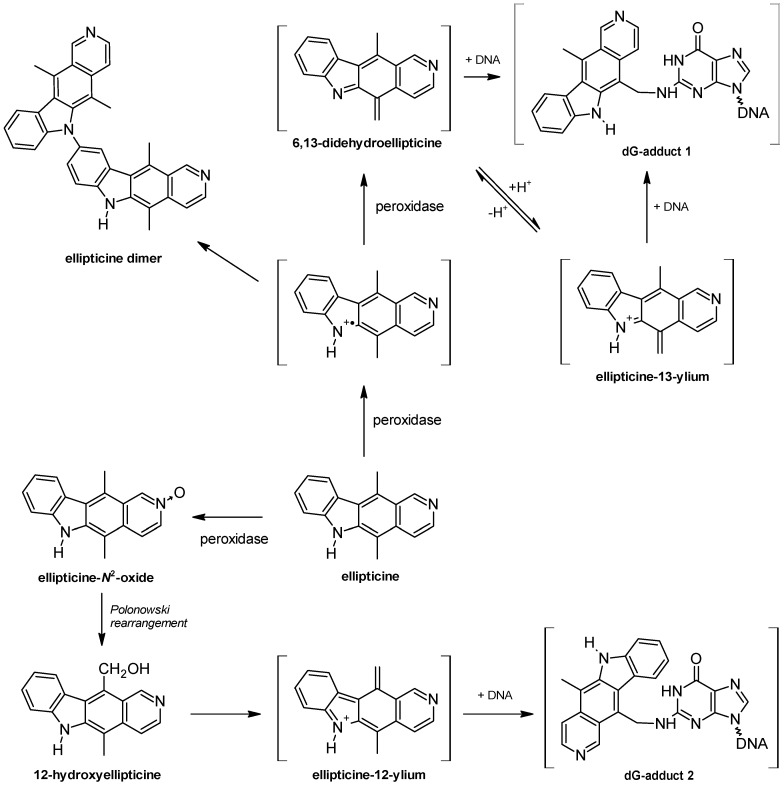
Oxidation of ellipticine by peroxidases showing the characterized metabolites and those proposed to form DNA adducts. The compounds shown in brackets were not detected under the experimental conditions and are the electrophilic metabolites postulated as ultimate arylating species or the postulated *N*²-deoxyguanosine adducts (adapted from reference [32]).

Besides CYP enzymes, peroxidases are able to oxidize ellipticine to metabolites generating covalent DNA adducts. Human myeloperoxidase, bovine lactoperoxidase, ovine cyclooxygenase (COX)-1, human COX-2 and plant horseradish peroxidase oxidize ellipticine *in vitro* to form up to four DNA adducts [32]. Even though mechanisms of oxidation of ellipticine by peroxidases and CYPs are different, two of the DNA adducts formed during oxidation of ellipticine by peroxidases are identical to those produced by 13-hydroxy- and 12-hydroxyellipticine generated by CYPs [32]. Ellipticine oxidation to 6,13-didehydroellipticine (the ellipticine methylene-imine) and ellipticine *N*^2^-oxide [32,59] explain the mechanisms of peroxidase-mediated formation of DNA adducts identical to those formed by CYPs (Figure 4). The two minor DNA adducts that are formed by peroxidases (spots 6 and 7 in Figure 2) [32], are also generated after ellipticine activation with hepatic microsomes from humans [31,60], rats [1,38,60], rabbits [1,39], and mice [48,61], and in several organs of mice (Figure 2E,F) and rats (Figure 2G) treated with ellipticine [33,37,48].

## 4. Oxidation of Ellipticine by CYP Enzymes *in Vivo*

In order to extrapolate from the *in vitro* data to the *in vivo* situation, additional factors have to be considered such as route of administration, absorption, renal clearance, and tissue-specific expression of enzymes metabolizing ellipticine. To identify CYP enzymes responsible for activation of ellipticine *in vivo*, several animal models were used: (I) wild-type (WT) and Hepatic P450 Reductase Null (HRN) mice; (II) the same mouse models, in which expression of enzymes of the mixed-function oxidase system was induced by benzo[a]pyrene (BaP); and (III) Wistar rats. The data obtained with these animal models revealed a paradox: namely, that CYP1A enzymes appear to be more important for activation of ellipticine *in vivo*, despite being involved in its metabolic detoxification *in vitro*.

### 4.1. Utilization of Wild-Type (WT) and Hepatic P450 Reductase Null (HRN) Mice to Identify Enzymes Metabolizing Ellipticine in Vivo

In HRN mice, POR, the most important electron donor to mouse CYPs, is deleted specifically in hepatocytes. This model was developed by Henderson *et al.* [62] to evaluate the role of both hepatic POR and CYPs in xenobiotic metabolism. Deletion of this enzyme results not only in the loss of essentially all hepatic CYP function, but also in the lack of direct reduction of xenobiotics by POR, an additional property of this enzyme. This mouse model has been used successfully to investigate the role of hepatic *versus* extra-hepatic CYP-catalyzed metabolism and the disposition of several carcinogens and drugs including ellipticine [48,62,63,64,65,66,67,68,69].

Using WT and HRN mouse lines, hepatic CYPs were demonstrated to be important in ellipticine-derived DNA adduct formation also *in vivo*, because up to seven ellipticine-specific DNA adducts were observed in liver, lung, kidney, spleen, colon and bladder (see Figure 2E,F for liver of WT and HRN mice). Deoxyguanosine adduct spots 1 and 2 derived from 13-hydroxy- and 12-hydroxyellipticine, respectively (Figure 2 and Figure 3), were the predominant adducts in all mouse tissues examined.

The finding that ellipticine-DNA adducts are formed in all organs tested in these animals suggest that ellipticine or its metabolites are distributed via the blood stream to different organs and that these tissues may have the metabolic capacity to oxidatively activate ellipticine. As found by Chadwick and co-workers [52,53] ellipticine is very rapidly distributed from the blood, and its excretion is essentially complete by 24 h in several species including mice, rats, dogs, and monkeys. The rate of ellipticine elimination from blood was found to reflect the rate of metabolism of this drug [52]. The main organ responsible for its biotransformation was found to be the liver, forming predominantly 9-hydroxyellipticine, which is excreted mainly in bile as its glucuronide or sulfate conjugate [52,53]. Other *in vivo* pathways involving hydroxylation at as yet unknown positions in the molecule have also been found [52,53]. As mentioned above, in *in vitro* experiments, ellipticine is oxidized by CYPs in hepatic microsomes from a variety of species, including humans, rats, rabbits and mice [31,39,48,49,60,61] to several hydroxylated derivatives, with 9-hydroxy-, 12-hydroxy- and 13-hydroxyellipticine as the major metabolites in most species. However, because 13-hydroxy- and 12-hydroxyellipticine are reactive and have been found to form the two major ellipticine-DNA adducts [31,32,39,47], they will not be easily detectable *in vivo*. In addition, in these early studies radioactively labelled ellipticine was found to be deposited in a number of organs with the highest levels in the liver, followed by kidney, lung, intestine and spleen, and was located primarily in the nuclear fraction [52]. Our more recent data would suggest that covalent binding of ellipticine to DNA can explain this localization [32,33,48].

**Figure 5:**
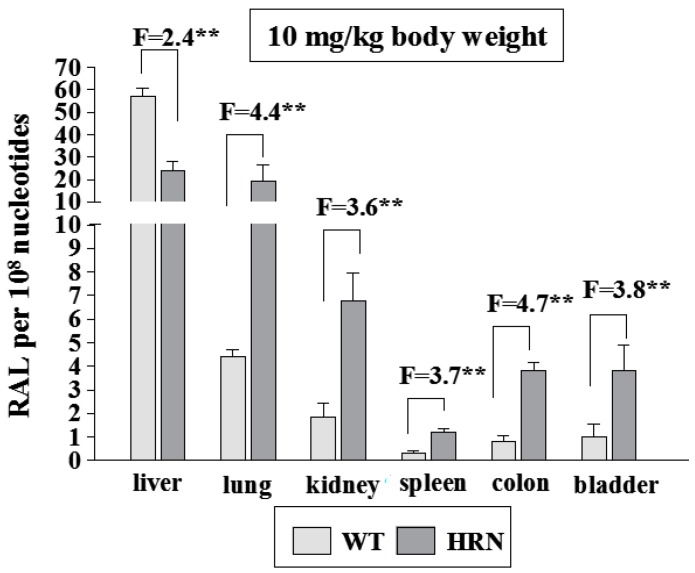
Total levels of ellipticine-DNA adducts determined and quantified by ^32^P-postlabelling analysis of DNA isolated from organs of HRN and WT mice treated *i.p.* with 10 mg ellipticine/kg body weight. F = fold higher and/or lower DNA adducts in HRN than WT mice. Columns, mean; bars, SD (*n* = 3); each DNA sample was analysed twice. ** *p* < 0.01. RAL, relative adduct labeling.

These tissues therefore have the metabolic capacity to oxidize ellipticine and, more importantly, the same reactive species forming DNA adducts are produced as in the liver, probably by both CYP catalysis and maybe peroxidases. The levels of ellipticine-DNA adducts in the livers of HRN mice were lower (by up to 65%) than those in WT mice, demonstrating that CYP enzyme activity is important for the oxidative activation of ellipticine to metabolites generating these adducts. Whereas hepatic CYP-mediated ellipticine DNA binding was reduced in HRN mice, adduct levels in extrahepatic organs were up to 4.7-fold higher (Figure 5).

Experiments utilizing *ex vivo* incubations of ellipticine with hepatic microsomes of WT and HRN mice and those of these mice exposed to BaP, as well as employing inhibitors of the most important CYP enzymes catalyzing detoxification and activation of ellipticine *in vitro*, CYP1A and 3A, respectively, helped to resolve which of these CYPs play a role in the mouse models. As expected, treatment of WT and HRN mice with BaP significantly induced expression of CYP1A, predominantly of CYP1A1 in liver (up to 175-fold), both at the transcriptional and translation levels [61,65]. This carcinogen also increased the expression of POR protein and its enzymatic activity in livers of these mouse models, but to a much lower extent, up to 2.9-fold. More interestingly, exposure of WT and HRN mice to BaP was also found to result in an increased expression of cytochrome *b*_5_, a protein of the microsomal mixed-function-oxidase system, in livers of these mice [66].

**Figure 6:**
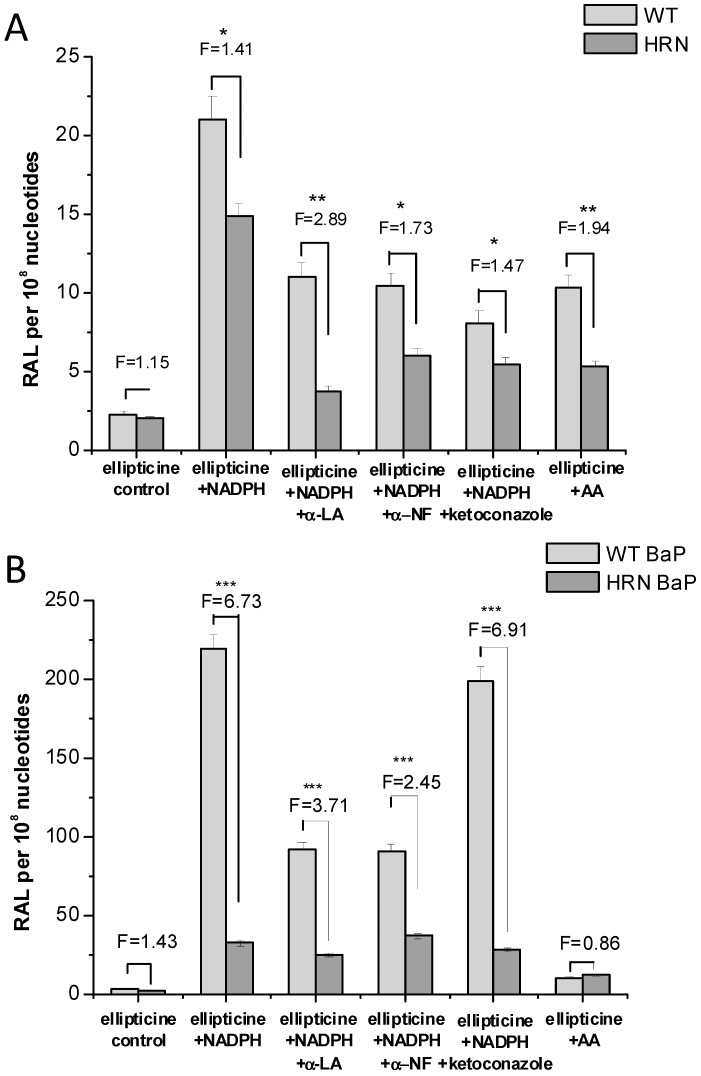
DNA adduct formation by ellipticine activated with microsomes isolated from livers of untreated Hepatic Cytochrome P450 Reductase Null (HRN) or wild-type (WT) mice (**A**) and from mice treated with BaP (**B**) as determined by ^32^P-postlabeling. F = fold higher DNA adducts levels in microsomes from WT mice compared to HRN mice. Columns: Mean RAL (relative adduct labeling) ± standard deviations (SD) shown represents total levels of DNA adducts of four determinations (duplicate analyses of two independent *in vitro* incubations). Values significantly different from HRN mice: * *p* < 0.05, ** *p* < 0.01, *** *p* < 0.001. Control = without cofactor; AA = arachidonic acid; α-NF = α-naphthoflavone; α-LA = α-lipoic acid.

In the presence of NADPH, a cofactor of POR- and CYP-dependent enzyme systems, the *ex vivo* incubations with ellipticine, DNA and hepatic microsomes of untreated (control) WT and HRN mice and mice treated with BaP led to activation of this drug to ellipticine-derived DNA adducts (Figure 2A,B and Figure 6), confirming the role of CYPs in ellipticine activation. Arachidonic acid, a cofactor for COX-dependent oxidation [48,64,70,71,72], also mediated formation of ellipticine-DNA adducts 1 and 2 in hepatic microsomes of all mice used. This suggests that COX also activates ellipticine in mouse liver [48,61], but arachidonic acid as a cofactor was much less effective than NADPH (Figure 6).

Surprisingly, levels of ellipticine-derived DNA adducts formed in the *ex vivo* incubations of HRN mice liver microsomes with NADPH were only 1.4-fold lower than amounts formed by hepatic microsomes from WT mice (Figure 6), even though POR expression in livers of HRN mice was two orders of magnitude lower. This finding indicates that ellipticine activation should be at least partially catalyzed also by enzymes with POR-independent activity [48]. Beside peroxidases that were found to activate ellipticine [32], the CYP2S1 enzyme, which is abundantly expressed in several tissues [73,74,75,76], might be such an enzyme, because it catalyzes the oxidation of compounds having polycyclic aromatic structures similar to ellipticine without participation of POR [75,76]. Whereas a role of a COX peroxidase in hepatic microsomes of WT and HRN mice was proven (see Figure 6) [48,61], the participation of CYP2S1 in ellipticine activation awaits further examination. Therefore, the human recombinant CYP2S1 enzyme heterologously expressed in *Escherichia coli* was prepared in our laboratory [77] and will be utilized to investigate efficiency of this CYP in ellipticine oxidation in an additional study.

At least two adducts (spots 1 and 2 in Figure 2A,B), which were identical to those generated *in vivo* in mice treated with ellipticine (Figure 2E,F) were formed by mouse hepatic microsomes. Furthermore, ellipticine-derived DNA adduct, spot A, was found as a minor adduct (Figure 2A), predominantly in microsomes isolated from HRN mice [48]. In incubations containing hepatic microsomes of WT and HRN mice treated with BaP, an additional adduct spot, corresponding to the 10-(deoxyguanosin-*N*^2^-yl)-7,8,9-trihydroxy-7,8,9,10-tetrahydrobenzo[*a*]pyrene (dG-*N*^2^-BPDE) adduct of BaP-7,8-dihydrodiol-9,10-epoxide with DNA *in vitro* and *in vivo* [65,78] was also detected (Figure 2B). This finding indicates that residual BaP is present in microsomes isolated from livers of WT and HRN mice treated with BaP, and is activated by CYP1A1 in combination with microsomal epoxide hydrolase to form this adduct.

Ketoconazole, a selective inhibitor of CYP3A [79,80], inhibited formation of ellipticine-DNA adducts in hepatic microsomes of untreated (control) WT and HRN mice, by ~60% (Figure 6A), confirming a role of CYP3A in ellipticine activation in mouse liver. However, the effect of this inhibitor was much lower in hepatic microsomes of BaP-treated WT and HRN mice, only by ~10% (Figure 6B). In mice exposed to BaP the contribution of CYP3A is much lower, because of the massive CYP1A induction by BaP. Surprisingly, this increased level of CYP1A, the enzymes that mainly detoxify ellipticine *in vitro*, led to higher amounts of ellipticine-DNA adducts formed (Figure 6), predominantly of adduct 1 [61]. Moreover, a selective inhibitor of CYP1A activities, α-naphthoflavone (α-NF) [79], inhibited formation of ellipticine-DNA adducts in all mice except HRN mice exposed to BaP (Figure 6). Induction of CYP1A in HRN mice by BaP also resulted in increased levels of ellipticine-DNA adducts, but α-NF caused an increase rather than a decrease in formation of ellipticine-DNA adducts (Figure 6). Therefore, here the BaP induced CYP1A enzymes seem to increase ellipticine detoxification.

**Figure 7:**
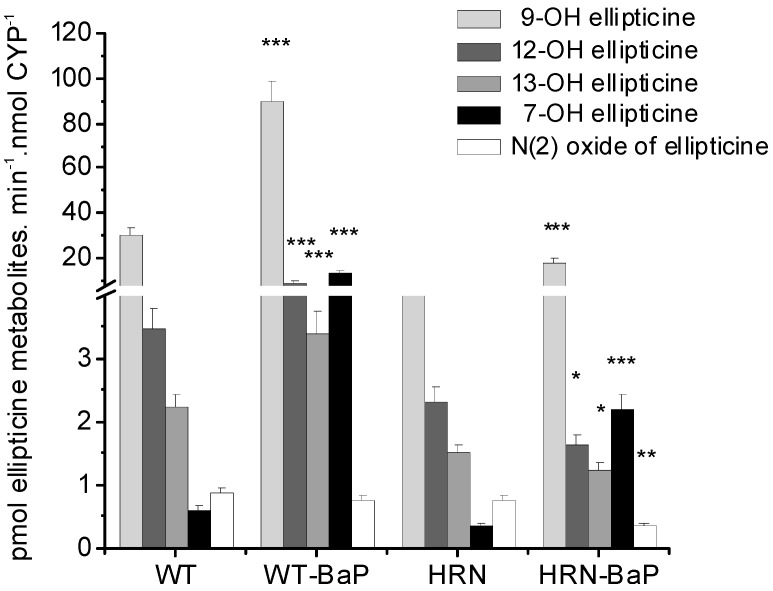
Levels of ellipticine metabolites formed by hepatic microsomes (0.2 mg protein) of Hepatic Cytochrome P450 Reductase Null (HRN) and wild-type (WT) mice from 10 μM ellipticine and by hepatic microsomes of HRN and WT mice pre-treated with BaP. Levels of ellipticine metabolites were determined by high performance liquid chromatography (HPLC) [31,60] and are averages ± standard deviations of triplicate incubations. Values significantly different from untreated mice: * *p* < 0.05, ** *p* < 0.01, *** *p* < 0.001.

Ellipticine metabolites formed in hepatic microsomes from all mouse lines used in previous studies [48,61] were analogous; 9-hydroxy-, 12-hydroxy-, 13-hydroxy, 7-hydroxyellipticine and *N*^2^-oxide of ellipticine were formed (Figure 7). However, the patterns of individual metabolites in WT and HRN mice, either control (untreated) or treated with BaP, were different. In incubations with HRN microsomes from untreated mice, 9-hydroxyellipticine levels were only one sixth, while the amounts of 13-hydroxy- and 12-hydroxyellipticine, were about one half of the levels in incubations with WT microsomes.

Exposure of mice to BaP induced CYP1A and resulted, as expected, in an increase in formation of 9-hydroxy- and 7-hydroxyellipticine (Figure 7). This result is consistent with previous studies where CYP1A1 and 1A2 were the major enzymes forming these metabolites [31,35,40]. Treatment of WT mice with BaP, however, also resulted in up to 2.5-fold higher levels of 13-hydroxy- and 12-hydroxyellipticine (Figure 7), the metabolites that were found to be formed *in vitro* mainly by CYP3A, and much less efficiently by CYP1A1 [31,39]. Hence not only CYP3A, but also CYP1A expressed in mouse liver are important for activation of ellipticine to ellipticine-DNA adducts in WT mice only, while in HRN mice the detoxification only is induced by BaP explaining the DNA adduct levels. These findings show that in contrast to the pure *in vitro* CYP systems (CYPs reconstituted with POR), CYP1A enzymes are responsible for ellipticine activation to form DNA adducts in mouse liver [48,61].

### 4.2. Ellipticine Metabolism in Wistar Rats

In order to further identify CYP enzymes responsible for activation and detoxification of ellipticine *in vivo*, Wistar rats were used. Also in this animal model, ellipticine treatment resulted in ellipticine-derived DNA adduct generation in several healthy organs (liver, kidney, lung, spleen, breast, heart and brain) (see Figure 2G for rat liver) [3,33,37] and in DNA of mammary adenocarcinoma [3]. The levels of ellipticine-derived DNA adducts generated in these adenocarcinomas were almost 2-fold higher than in normal healthy mammary tissue. This finding indicates that other CYPs such as CYP1B1, able to activate ellipticine may be expressed at higher levels in this adenocarcinoma [41] than in peritumoral tissues. Indeed, we [41], and others [81,82,83] previously showed CYP1B1 to be a typical CYP expressed in breast cancer.

Genotoxic side effects of ellipticine in healthy organs of experimental animals *in vivo* may of course also be determined by expression levels of CYP enzymes activating ellipticine in these tissues [33,37,48]. Indeed, several studies have found a positive correlation between DNA adduct levels of carcinogens or genotoxic agents, their persistence and their mutagenicity and/or tumorigenicity [84,85,86,87,88]. To better understand the role of ellipticine-DNA adducts in genotoxic side effects in healthy tissues, we have analyzed the persistence of ellipticine-DNA adducts in liver, lung, kidney, spleen, heart, and brain of rats to model the bioactivation of ellipticine in humans treated with ellipticine [33]. Only very low levels of adducts persisted only in some tissues. In addition, not all ellipticine-DNA adducts persist in the tissues analyzed in the study (only adducts 1, 2, 4, and 5) [33]. This finding demonstrates that healthy tissues of rats treated with ellipticine possess effective DNA repair systems to remove certain lesions and suggests a relatively low impact of the genotoxic side effects of ellipticine during cancer treatment in humans.

Also in rats, formation of ellipticine-DNA adduct 1 is dependent not only on levels of CYP3A, but also on those of CYP1A1. The levels of ellipticine DNA adduct 1 in analyzed organs correlated not only with expression levels of CYP3A, but also with those of CYP1A1 in the same organs, which again does not correspond to the situation *in vitro*. As outlined above, in the *in vitro* systems, CYP3A is mainly responsible for formation of ellipticine-DNA adducts and CYP1A predominantly oxidizes ellipticine to its detoxification metabolites [3,4,36]. *In-vitro* studies investigating the effect of cytochrome *b*_5_ on the metabolism of ellipticine explained the discrepancies between CYP oxidation of ellipticine *in vitro* and *in vivo*, because this protein has a crucial role in directing individual CYP enzymes to ellipticine activation or detoxification. Moreover, the induced expression of cytochrome *b*_5_ protein in liver of rats treated with ellipticine [35,51] suggests that cytochrome *b*_5_ may modulate the CYP-mediated bioactivation and detoxification of ellipticine in this animal model *in vivo* as well.

Cytochrome *b*_5_ is an important component of the microsomal mixed-function-oxidase system and can influence the metabolism of xenobiotics [69,78,89,90,91,92,93,94,95]. For more than four decades, the role of cytochrome *b*_5_ in CYP catalysis has been controversial, and based entirely on *in vitro* data, which showed that cytochrome *b*_5_ could inhibit or stimulate CYP activity depending on a number of variables including CYP isoenzyme, substrate and cytochrome *b*_5_ concentration [89,90,91,92,93,94]. In order to investigate the role of cytochrome *b*_5_ in ellipticine metabolism we conducted some *in vitro* experiments using human liver microsomes, hepatic microsomes from control and ellipticine-pretreated rats and reconstituted systems with human CYP1A1, CYP1A2, CYP3A4, POR, and cytochrome *b*_5_ in different ratios [34,35,39,49,51,96]. We found that cytochrome *b*_5_ alters the ratio of ellipticine metabolites generated by CYP1A1, 1A2, and 3A4. Whereas the amounts of the detoxification metabolites (7-hydroxy- and 9-hydroxyellipticine) are either decreased (CYP1A1/2) or not changed (CYP3A4) with cytochrome *b*_5_ added to the reconstituted system, the amounts of the active metabolites, 12-hydroxy- and 13-hydroxyellipticine, increased considerably, leading to higher ellipticine-DNA adduct levels [34,35,39].

### 4.3. Studies with Human Microsomes and Cancer Cells

Because in the studies described above with isolated enzymes the ratios of the various partners of CYP are generated experimentally, a more physiological model to identify human enzymes responsible for ellipticine activation was used, namely, human hepatic microsomes [31,39]. These microsomal fractions contain a mixture of human CYPs, POR, cytochrome *b*_5_ and its reductase (NADH:cytochrome *b*_5_ reductase). Thus, they comprise the essential components of the enzymatic system metabolizing drugs, mimicking well a situation in human liver, where a majority of drug metabolism occurs [78,95]. Human hepatic microsomes oxidize ellipticine mainly to 13-hydroxy- and 12-hydroxyellipticine, whereas 7-hydroxy-, 9-hydroxyellipticine and ellipticine *N*^2^-oxide are generated at more than 10-fold lower amounts (see table 2 in [39]). Similar results were found in hepatic microsomes of rats [60]. As a consequence, high levels of both major ellipticine-DNA adducts are formed when DNA is added to the microsomal incubations. The amounts formed correlated with the activity of the major CYPs found previously to form the metabolites generating these DNA adducts (see above) [31,39].

All these results explained why the CYP1A enzymes are more important in ellipticine activation *in vivo*; their activity is modulated by cytochrome *b*_5_. These results also demonstrated that not only the expression levels of CYP1A and 3A in several species including human, but also the amounts of expressed cytochrome *b*_5_ dictate the oxidative activation and detoxification of ellipticine *in vivo*. Moreover, because ellipticine itself is capable of inducing expression of CYP1A (predominantly CYP1A1), POR, and cytochrome *b*_5_ [51,96], it increases its own metabolism leading predominantly to activation of this drug to reactive species forming DNA adducts [34,35], thereby modulating its own pharmacological potential.

The ellipticine-derived DNA adducts formed by enzymatic activation of ellipticine *in vitro* and *in vivo* were also found in human tumor cells, in which CYP enzymes are expressed. The adducts were found in human adenocarcinoma MCF-7 [41], neuroblastoma IMR-32, UKF-NB-3, and UKF-NB-4 [43,44], glioblastoma U87MG [46], and BHT-101, B-CPAP and 8505-C thyroid cancer cells [45] exposed to ellipticine. Cytotoxicity of ellipticine corresponded to the amounts of ellipticine-DNA adducts formed in the specific cancer cells and depended on expression levels of CYP enzymes metabolizing ellipticine (CYP1A1, 1B1, and 3A4) and/or cytochrome *b*_5_ in these cells [36,41,43,44,45,46]. High expression levels of cytochrome *b*_5_ together with those of CYP1A1 and 3A4 lead to more ellipticine-DNA adducts and higher cytotoxicity of ellipticine predominantly in neuroblastoma UKF-NB-4, glioblastoma U87MG and thyroid cancer cells [14,36,41,43,44,45]. These findings again demonstrate the importance of expression of CYPs, but also of cytochrome *b*_5_, in the tumor and normal tissue, because the ratios of these enzymes determine the pharmacological effects of ellipticine.

## 5. Conclusions

The data summarized in this review demonstrate that the DNA-damaging anticancer alkaloid ellipticine might be considered a prodrug, whose major mechanism of action is mediated by an enzymatic activation leading to formation of covalent DNA adducts in target tissues. These ellipticine-DNA adducts are formed in both healthy and tumor tissues and cells, but they do not persist in healthy tissues. The data show that cytotoxic effects of ellipticine in tumor tissues are dictated by (I) levels of CYP expression (and/or peroxidase expression); (II) levels of cytochrome *b*_5_ expression; and (III) its own potency to induce CYP1A1, CYP3A and cytochrome *b*_5_ in tumor tissues and cells. The results also demonstrate that animal models, where levels of biotransformation enzymes either knocked out or induced, are appropriate tools to identify enzymes responsible for the metabolic activation and detoxification of ellipticine. They also demonstrate that extrapolation from *in vitro* data to the situation *in vivo* is not always possible, confirming the need for these animal models.

Even though the role of cytochrome *b*_5_ in modulation of ellipticine metabolism *in vitro* was clearly shown, its effect *in vivo* is still quite enigmatic. Two mouse lines, one with a conditional hepatic deletion of cytochrome *b*_5_ (HBN, Hepatic cytochrome *b*_5_ Null) [97] and a double conditional mutant, HBRN (Hepatic cytochrome *b*_5_/P450 Reductase Null), in which both enzymes are deleted specifically in the liver [98], which were recently developed in the laboratory of Wolf and coworkers [97,98], may help to resolve the role of cytochrome *b*_5_ in CYP-mediated metabolism of ellipticine *in vivo*.

The results summarized in this review form the basis to further predict the susceptibility of human cancers to ellipticine and suggest this alkaloid for treatment in combination with CYP gene transfer (CYP-gene-directed enzyme-prodrug therapy) [99,100], which has the potential to provide efficient activation of ellipticine in target tumor tissue, thereby increasing the anticancer potential of this prodrug. Furthermore, two of the ellipticine metabolites formed by oxidation with CYPs in combination with cytochrome *b*_5_, 13-hydroxy- and 12-hydroxyellipticine, are reactive enough to decompose spontaneously to the carbenium ions forming DNA adducts that are predominantly responsible for killing cancer cells. Both these ellipticine metabolites are, therefore, excellent candidates for tumor-specific targeting by appropriate derivatives, including their encapsulated forms into nanocarriers. Research into such targeted carrier systems for active ellipticine metabolites is a major research aim on the path to clinical application of ellipticine in tumor therapy.
